# SOS System Induction Inhibits the Assembly of Chemoreceptor Signaling Clusters in *Salmonella enterica*

**DOI:** 10.1371/journal.pone.0146685

**Published:** 2016-01-19

**Authors:** Oihane Irazoki, Albert Mayola, Susana Campoy, Jordi Barbé

**Affiliations:** Departament de Genètica i de Microbiologia, Universitat Autònoma de Barcelona, Eix Central, Edifici C, 08193, Bellaterra (Barcelona), Spain; University of Illinois at Urbana-Champaign, UNITED STATES

## Abstract

Swarming, a flagellar-driven multicellular form of motility, is associated with bacterial virulence and increased antibiotic resistance. In this work we demonstrate that activation of the SOS response reversibly inhibits swarming motility by preventing the assembly of chemoreceptor-signaling polar arrays. We also show that an increase in the concentration of the RecA protein, generated by SOS system activation, rather than another function of this genetic network impairs chemoreceptor polar cluster formation. Our data provide evidence that the molecular balance between RecA and CheW proteins is crucial to allow polar cluster formation in *Salmonella enterica* cells. Thus, activation of the SOS response by the presence of a DNA-injuring compound increases the RecA concentration, thereby disturbing the equilibrium between RecA and CheW and resulting in the cessation of swarming. Nevertheless, when the DNA-damage decreases and the SOS response is no longer activated, basal RecA levels and thus polar cluster assembly are reestablished. These results clearly show that bacterial populations moving over surfaces make use of specific mechanisms to avoid contact with DNA-damaging compounds.

## Introduction

Swarming is the rapid, flagellar-driven, and highly coordinated translocation of a bacterial colony across a moist surface [1]. This form of motility is widely distributed throughout the Domain *Bacteria*, in which it is associated with increased antibiotic resistance [2–4] and virulence [5–9]. In fact, swarming is one of the first steps in the bacterial colonization of host surfaces [8,10,11].

*Salmonella enterica*, the most frequent cause of food-borne disease outbreaks worldwide [12], is able to swarm on soft agar surfaces (0.5–0.8% agar) and is thus considered a temperate swarmer [13]. During swarming, the morphology of temperate swarmers does not significantly change, and processes such as elongation, the formation of multi-nucleoid cells, and hyperflagellation are not observed, unlike in robust swarmers such as *Proteus* and *Vibrio* species [14,15]. While swarming by *Salmonella* is clearly related to bacterial invasion and the expression of virulence factors [4,10,11,16,17], little is known about the mechanisms that control this form of motility. It is well established that the chemotaxis signaling pathway, but not chemotaxis itself, plays a key role in the swarming motility of *S*. *enterica* [18]. The chemotaxis pathway includes transmembrane ligand receptors, known as methyl-accepting chemotaxis proteins (MCPs), which interact with each other to form trimers of dimers that are associated, through CheW adaptor proteins, with the CheA kinase. These signaling complexes, present in bacterial cells in amounts ranging from a few to thousands of copies, normally cluster together at the cell poles, where they form signaling arrays [19–21]. During chemotaxis, signal recognition by chemoreceptors modulates CheA kinase autophosphorylation. In turn, phosphorylated CheA mediates phosphorylation of the CheY response regulator, which acts on the flagellar motor to prompt flagellar rotation switching [22,23]. Swarming, however, requires only flagellar propulsion and the related mechanical interactions; the fine control offered by the chemotaxis pathway is dispensable [24–26]. The flagellar switch promotes lubrication of the cell–surface interface, thus minimizing surface friction and allowing swarming motility by temperate swarmers [13]. Mutants with defects in the chemotaxis pathway, flagellar biosynthesis, or polar chemoreceptor cluster assembly give rise to non-swarming colonies [27–31].

The RecA protein is also related to swarming ability [32–34]. RecA is a multifunctional protein that during DNA damage stress acts as a positive regulator of the SOS system, which mediates DNA repair [35]. The SOS response comprises a genetic regulatory network that is widely distributed among *Bacteria*. When DNA damage occurs, the RecA protein acquires an active conformation (RecA*) that promotes autocleavage of the SOS system repressor (the LexA protein) and the SOS response induction [36]. After its autohydrolysis, the LexA repressor is no longer able to repress either its own expression or that of the genes it controls (including *recA*, which is also part of the SOS network), thereby inducing the SOS response [37]. Once the DNA lesions are repaired, RecA is no longer activated and LexA again represses expression of the genes directly involved in the SOS network, which restores their basal-level expression. The SOS response coordinates the expression of genes involved in DNA recombination, DNA repair, cell division inhibition, mutagenesis, pathogenesis, antibiotic resistance, biofilm formation, and mobile element distribution [38–42].

The absence of the RecA protein impairs the swarming ability of both *Escherichia coli* and *S*. *enterica* [33,34]. We recently reported that, at least in *S*. *enterica*, this defect occurs because the RecA protein is essential for standard flagellar rotation switching and for the formation of normal chemoreceptor polar arrays [32]. Moreover, not only the absence but, conversely, also the overexpression of RecA in the absence of DNA damage impedes swarming motility. We were thus able to show that a *recA*-overexpressing mutant of *S*. *enterica* has both a non-swarming phenotype and a significantly reduced capacity to cross the intestinal epithelium [43]. In both the absence and the overexpression of RecA, a link between the RecA protein and the chemotaxis pathway, through the CheW anchor protein, has been suggested [32,34]. In fact, the interaction between RecA and CheW was confirmed by co-immunoprecipitation assays [32]. Furthermore, it has been demonstrated that the restoration of a normal swarming phenotype in a *recA*-overxepressing strain can be achieved by increasing the concentration of intracellular CheW [34].

Despite these insights, the effect of SOS response induction on swarming and the pathways by which increased RecA levels inhibit swarming have yet to be determined. The aim of this work was to further dissect these mechanisms in order to deepen our understanding of how bacterial cells adapt to a surface niche and respond to external stimuli. Specifically, we studied swarming ability and chemoreceptor polar cluster assembly in *S*. *enterica* in the presence of the SOS system inducer mitomycin C and the roles played by CheW and RecA proteins. We found that induction of the SOS response impairs swarming motility by reversibly bypassing chemoreptor polar array assembly, through a disturbance of the balance between RecA and CheW.

## Materials and Methods

### Bacterial strains, plasmids, and growth conditions

The bacterial strains and plasmids used in this study are listed in S1 Table. Except when indicated, all strains were grown at 37°C in Luria–Bertani (LB) broth or on LB plates. When necessary, ampicillin (100 μg/ml), kanamycin (100 μg/ml), and/or chloramphenicol (34 μg/ml) were added to the culture. The growth conditions for swarming and the polar cluster assays are described elsewhere in this section.

The vectors used in this work are also listed in S1 Table. The molecular procedures required for this work were described previously [44]. *E*. *coli* DH5α strain was used in vector constructions. When needed, vectors were transformed in the corresponding *S*. *enterica* or *E*. *coli* strain by electrotransformation.

### Construction of the *S*. *enterica* mutant strains

The *S*. *enterica cheW* FLAG-tagged mutant was constructed as described previously [45] using the pKO3 plasmid [46]. An overlap-extension PCR-generated *cheW*::*FLAG* gene fusion (which adds the DYKDDDDK epitope to the CheW protein) was introduced at the *Bam*HI restriction site of pKO3, generating plasmid pUA1121. The vector was confirmed by sequencing and electroporated into *S*. *enterica* ATCC14028. The resulting mutants were confirmed by sequencing and western blot. One mutant (UA1916) was selected for further studies.

To construct *S*. *enterica recAo cheW*::*FLAG*, the marker *recAo6869* was introduced into UA1916 strain by transduction using the P22int7(HT) bacteriophage [47] and UA1876 as the donor strain [34]. The absence of the prophage in the transductants was determined by streaking them onto green plates as described previously [48]. The resulting strains were verified by sequencing and the deregulation of *recA* expression was confirmed by ELISA, using the anti-RecA antibody (see below). The same procedure was used for the *ΔcheR* mutant derivatives, generated using the P22 int7(HT) bacteriophage and UA1907 strain. The latter includes the *ΔcheR* construct obtained by one-step PCR-based gene replacement and the chloramphenicol resistance cassette from pKD3 instead of the native *cheR* gene [49].

The *S*. *enterica* ATCC14028 *ΔsulA* mutant was constructed by one-step PCR gene replacement as described previously [49,50].

Gene substitution in all constructs was confirmed by PCR using the appropriate primers followed by sequencing.

### Swarming assays

Swarming motility was assayed as described previously [33,34] using the corresponding *S*. *enterica* strains (S1 Table). Briefly, freshly prepared LB-swarming plates (1% tryptone, 0.5% yeast extract, 0.5% NaCl, 0.5% d-(+)-glucose, and 0.5% agar) supplemented when needed with suitable antibiotics, IPTG, and/or mitomycin C (0.08 μg/mL) were point inoculated using a sterile toothpick with a single *S*. *enterica* colony of the corresponding strain grown on normal LB plates. Once inoculated, the plates were incubated at 37°C for 14 h, by which time the wild-type strain had grown to reach the plate borders.

The same procedure was used for the swarming assays in the presence of a mitomycin C gradient generated by the disk diffusion method, as described previously [51]. Sterile filter-paper disks (Whatman 6 mm, grade AA discs, GE) were soaked in either water or mitomycin C (2 mg/mL), dried at room temperature for 2 h, and aseptically placed onto the LB-swarming plates. The plates were then inoculated with the corresponding strains as described above and incubated at 37°C. Bacterial migration was observed for the indicated time.

To evaluate swarming motility, the plates were photographed (ChemiDoc XRS + system, Bio-Rad) and the diameter of the swarming colony was measured using ImageJ software (National Institutes of Health). The swarming ability of each strain under each condition was determinate at least three times, each in triplicate. The images shown in the figures are representative of the entire image set.

The relative swarming motility index (RSMI) for each condition was calculated as the ratio of the colony diameter of the studied strain to that of the control strain under the same experimental conditions, as described previously [52].

### Mitomycin C cell susceptibility assay

To evaluate the cell susceptibility to mitomycin C, the corresponding bacterial inoculum was applied using a sterile swab all over the surface of LB plates. Afterwards, disks soaked in mitomycin C (2 mg/mL), prepared as described above, were placed onto the middle of the inoculated plates. After 14h of incubation, the bacterial growth inhibition zone was observed.

The plates were photographed (ChemiDoc XRS + system, Bio-Rad) and the diameter of the cell growth inhibition was measured using ImageJ software (National Institutes of Health). The images shown in the figure are representative of the entire image set.

### Chemoreceptor clustering assay

*S*. *enterica ΔcheR* strains carrying the pUA1127 vector containing the *eYFP*::*cheR* fusion under the control of a IPTG-inducible promoter (P*tac*) were used (S1 Table) to visualize the polar, round, diffraction-limited spots previously referred to as polar clusters [53]. The eYFP::CheR fusion served as a polar cluster localization reporter, as described previously [27,29,32,53]. In these strains, the *cheR* gene was removed to better visualize the chemoreceptor arrays by avoiding the presence of native CheR protein. Clustering experiments were performed as described previously [32], except that in this work the corresponding strains were grown, depending on the experiment, on LB-swarming plates or in liquid medium. In the former, samples were taken as described previously [54]. Briefly, the cells were grown on LB-swarming plates supplemented with ampicillin, 25 μM IPTG and, when needed, 0.08 or 10 μg mitomycin C/mL or 0.06 μg ciprofloxacin C/mL (final concentration). After 14 h of incubation at 37°C, the cells were suspended in 1 mL of ice-cold tethering buffer (10 mM potassium-phosphate pH 7, 67 mM NaCl, 10 mM Na-lactate, 0.1 mM EDTA, and 0.001 mM l-methionine) by gently tilting the plates back and forth and harvested by 15 min of low-speed centrifugation (5000 g). With this method, migrating cells were easily lifted off the surface, whereas the vast majority of cells in the middle of the plates remained intact on the surface. Non-swarming colonies were recovered using the same method but with 0.5 mL of cold tethering buffer.

For cells grown in liquid medium, overnight cultures of the corresponding *S*. *enterica* strains were grown under constant agitation at 30°C in tryptone broth (TB) supplemented with ampicillin and 25 μM IPTG. One day later, the cultures were diluted 1:100 in TB without antibiotics but with the addition of 25 μM IPTG to maintain the induction of the *eYFP*::*cheR* fusion construct. The cultures were incubated at 30°C until an OD_600_ of 0.08–0.1 was reached. Mitomycin C was then added to the corresponding culture to achieve a final concentration of 0.08 μg/mL or 10 μg/mL. The samples were collected at the indicated times and the cells were harvested by low-speed centrifugation for 15 min. For reversibility studies, cultures treated for 300 min were harvested by centrifugation. The supernatant was discarded and the cells were resuspended in TB with or without the SOS inducer and incubated at 30°C. Samples were collected at the indicated times and the cells were harvested by low-speed centrifugation for 15 min.

In all experiments, the harvested cells were washed once using ice-cold tethering buffer, resuspended in 20–100 μL of the same buffer, and maintained on ice until they were applied onto thin 1% agarose pads as described previously [32].

Fluorescence microscopy was performed using a Zeiss AxioImager M2 microscope (Carl Zeiss Microscopy) equipped with a Zeiss AxioCam MRm monochrome camera (Carl Zeiss Microscopy) and a filter set for eYFP (excitation BP500/25; beam splitter FT 515; emission BP535/30). Cell fields were photographed and at least 500 cells were visually inspected to determine the presence and type of clusters in each sample. All images were acquired under identical conditions. Each experiment was performed at least in triplicate using independent cultures; a minimum of 1500 cells from each studied strain of *S*. *enterica* were therefore analyzed. The images presented in the figures are representative of the entire image set. ImageJ software (National Institutes of Health) was used to quantify the number of clusters and to prepare images for publication.

### ELISA for CheW and RecA quantification

Samples for the ELISA were obtained either by recovering the cells directly from the colony edge of the corresponding LB-swarming plates, following the same procedure as described above, or by sampling the culture at the same time that it was used in a polar cluster assay. In both cases, cells were resuspended in sonication buffer (PBS 1×, cOmplete mini EDTA-free tablets, pH 7.3) and whole-cell lysates were obtained by sonication (2 30-s pulses and 20% amplitude, Digital sonifierR 450, Branson). After centrifugation (12000 g for 10 min), the supernatants were recovered and the total protein concentration of each sample was quantified according to the Bradford method using the protein reagent DyeR (BioRad) and a bovine serum albumin standard curve (range: 1.5–200 μg/mL).

The RecA and CheW::FLAG proteins used in the standard quantification curves were cloned in overexpression vectors, purified using *E*. *coli* BL21 (DE3) strain pLysS, and overexpressed by the addition of IPTG to the cultures. The *recA* gene was cloned in the pGEX-4T-1 vector, which includes a GST-tag (pUA1125), and purified by glutathione affinity chromatography using Sepharose 4BR resin (GE Healthcare) following the manufacturer’s instructions. The *cheW*::*FLAG* gene was inserted into the pET15b overexpression vector (Novagene); the protein products were purified using the Talon kit (Clontech). The RecA and CheW::FLAG proteins were eluted by the addition of 20 μL of a solution containing 1 U thrombin/μL. The final concentrations of the two proteins were quantified using the Bradford method as described above.

RecA and CheW::FLAG proteins were quantified by ELISA as described [55]. Pre-treated 96-well microtiter plates (Nunc-Immunoplate F96 Maxisorp, Nunc) were coated with serial dilutions of the whole-cell lysates. Purified RecA and CheW::FLAG proteins were used for the standard quantification curve, and lysates from a *S*. *enterica ΔrecA* strain [55] and *S*. *enterica* wild-type as background controls for RecA and CheW::FLAG quantifications, respectively. These controls were necessary to correct for possible unspecific binding of the antibodies to other cellular components of the lysates. Anti-RecA (monoclonal antibody to ARM193 RecA clone, MBL) and anti-FLAG (monoclonal antibody to DYKDDDDK epitope Tag, Acris) mouse IgG antibodies were used in RecA and CheW::FLAG quantification. The secondary antibody was an anti-mouse-IgG horseradish-peroxidase-conjugated antibody (polyclonal antibody to mouse IgG (HEL)-HRP, Acris). The BD OptEIA TMB substrate reagent set (BD Biosciences), prepared following the manufacturer’s instructions, was used as the developing solution. Plate measurements were made at 650 nm using a multiplate reader (Sunrise, Tecan).

### Statistical analysis

The results of the chemoreceptor clustering assay were statistically evaluated using a one-way analysis of variance (ANOVA) with Prism (GraphPad), as previously described [32,56,57]. The analyses were followed by the Bonferroni multiple comparison post-hoc test. A *p* value <0.05 was considered to indicate statistical significance. In all cases, the error bars in the figures indicate the standard deviation.

## Results

### *S*. *enterica* swarming ability and SOS system induction

**Fig 1:**
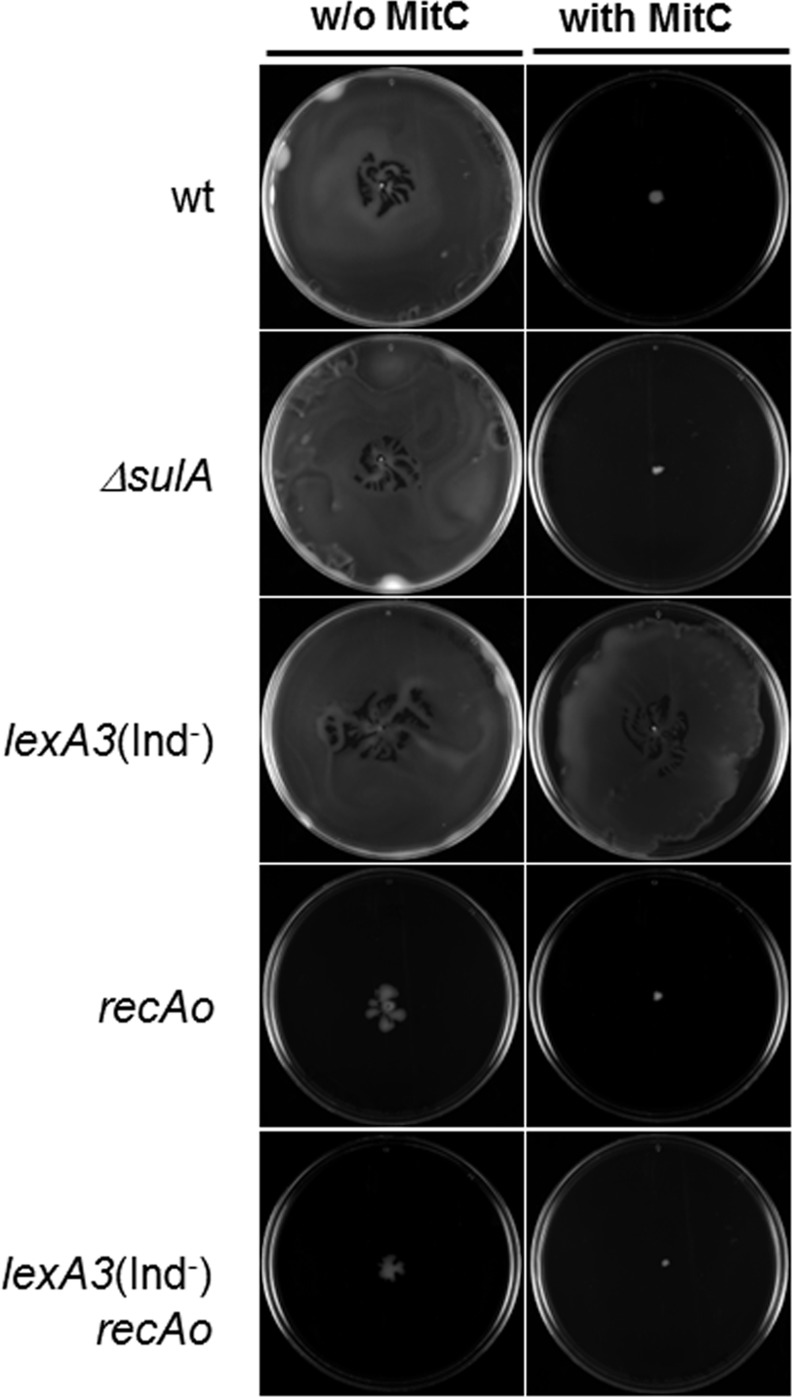
Swarming ability of the *S*. *enterica* wild-type and the *ΔsulA*, *recAo*, *lexA3*(Ind^−^), and *lexA3*(Ind^−^) *recAo* mutant derivatives in the absence or presence of mitomycin C. Representative images of a bacterial colony swarming on a semisolid agar surface following incubation of the culture for 14 h at 37°C. When indicated, 0.08 μg mitomycin C/mL was added to the semisolid agar plates.

The effects of the SOS system inducer mitomycin C on the swarming behavior of the wild-type strain and on four different SOS-network-mutants were analyzed. In the absence of mitomycin C, the wild-type, *lexA3*(Ind^−^) (containing a non-hydrolyzable LexA repressor [58]) and *ΔsulA* (lacking the SOS-associated cell division inhibitor SulA [59]) strains were able to swarm (Fig 1). The *recAo* (carrying a point mutation in its LexA operator resulting in the constitutive expression of *recA* [60]) and *recAo lexA3*(Ind^−^) mutants had a non-swarming phenotype either in the absence or presence of a sublethal concentration of mitomycin C (Fig 1). Swarming ability was also inhibited by the presence of mitomycin C in wild-type and *ΔsulA* strains but not in the *lexA3*(Ind^−^) mutant (Fig 1). These results clearly indicated that SOS response activation impairs swarming ability via a SulA-independent pathway, since in the presence of mitomycin C neither the wild-type and nor the *ΔsulA* strain swarmed. Moreover, the absence of swarming motility in the wild-type, *ΔsulA*, *recAo*, and *recAo lexA3*(Ind^−^) strains cultured in the presence of mitomycin C was not due to any substantial decrease in cell viability since swarming was exhibited by the *lexA3*(Ind^−^) mutant cultured under the same conditions (Fig 1). Finally, the *recAo lexA3(*Ind^−^) strain (which is incapable of SOS response induction but expresses high levels of RecA) is unable to swarm, either in the absence or presence of mitomycin C indicating that the activation of RecA is not necessary for swarming inhibition. Taken together, these results show that among all the cellular-associated phenomena that make up the SOS response, only the amplification of RecA impairs swarming motility.

### Effect of SOS induction on chemoreceptor polar cluster assembly

The increase in RecA mediated by the SOS response generates the same non-swarming phenotype (Fig 1) exhibited by CheW-overexpressing strains [29]. High levels of CheW interfere with the assembly of trimers of chemoreceptor dimers, which prevents the formation of the polar chemoreceptor clusters by cells growing in liquid medium [27]. We therefore asked whether the increase in intracellular RecA levels that occurs during SOS system induction gives rise to the same defect in chemoreceptor polar cluster formation in swarming cells. To examine this possibility, we constructed *ΔcheR* mutant derivatives of the wild-type, *ΔsulA*, *recAo*, *lexA3*(Ind^−^), and *recAo lexA3*(Ind^−^) strains carrying the pUA1127 plasmid containing an *eYFP*::*cheR* fusion (S1 Table) and then analyzed the dynamics of chemoreceptor polar cluster assembly in swarmer cells in the presence of mitomycin C. The eYFP::CheR fusion was previously used as a reporter for polar cluster localization [27,29,32,53]. Note that neither *cheR* deletion nor the presence of the pUA1127 plasmid affected the swarming phenotype of the parental strains shown in Fig 1 (data not shown).

**Fig 2:**
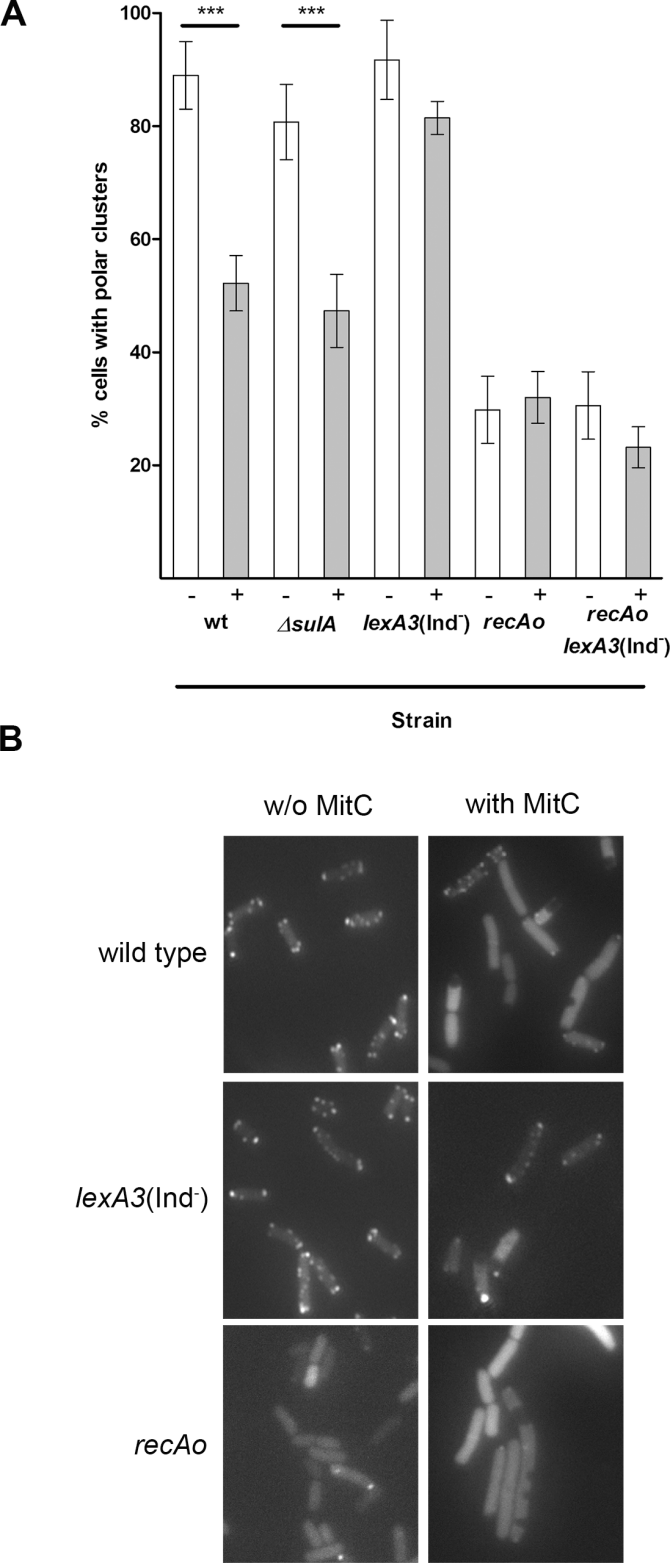
**A)** Percentage of cells of *S*. *enterica ΔcheR* harboring plasmid pUA1127 (wild type) and of *ΔsulA*, *recAo*, *lexA3*(Ind^−^) or *lexA3*(Ind^−^) *recAo* mutant derivatives that developed polar clusters while growing on swarming plates in the absence (-) or presence (+) of mitomycin C. The cells were harvested from the edge of the swarming colony growing on soft agar plates. When indicated, 0.08 μg mitomycin C/mL was added to the plates. The results are the mean of at least four independent imaging studies. Error bars represent the standard deviation. ***p<0.001 as determined by a one-way ANOVA with a Bonferroni correction. **B)** Representative fluorescence microscopy images of cells from wild-type, *lexA3*(Ind^−^), and *recAo* strains grown in the presence or absence mitomycin C.

The percentage of polar-cluster-containing cells growing on swarming plates with or without mitomycin C is shown in Fig 2. Polar clusters formed in >90% of wild-type cells grown in the absence of mitomycin C but in only about 50% of the cells grown in the presence of the SOS inducer. The same results were obtained in the *ΔsulA* strain. In the *lexA3(Ind*^−^) strain, either in the absence or presence of mitomycin C, the percentage of cells with polar clusters was almost 90, i.e., the same as in wild-type cells growing without mitomycin C. In the *recAo* and *recAo lexA3*(Ind^−^) strains only about 30% of the cells contained polar clusters, regardless of the presence or absence of mitomycin C (Fig 2). Thus, the inability of the cells to form polar clusters correlated with the non-swarming phenotype (Fig 1). The same association was reported in studies associated to *cheW* and *Δtol pal E*. *coli* mutants [27,29].

### Temporal evolution of polar chemoreceptor cluster assembly during SOS response induction

To further understand the changes in polar chemoreceptor cluster assembly originated by mitomycin C treatment, we evaluated the percentage of polar-cluster-containing cells as well as RecA protein concentrations during SOS system induction. In addition, since CheW overexpression gives rise to a decrease in polar arrays [27], we measured the CheW concentration in mitomycin-C-treated cells, although the *cheW* promoter does not contain a LexA operator [61,62]. As there are no commercial antibodies against CheW, a FLAG-tag was added to the *cheW* gene of the *S*. *enterica ΔcheR* harboring the eYFP::CheR fusion (pUA1127). The FLAG-tag did not change the swarming phenotype of this strain (data not shown) but it did allow CheW quantification during SOS induction.

**Fig 3:**
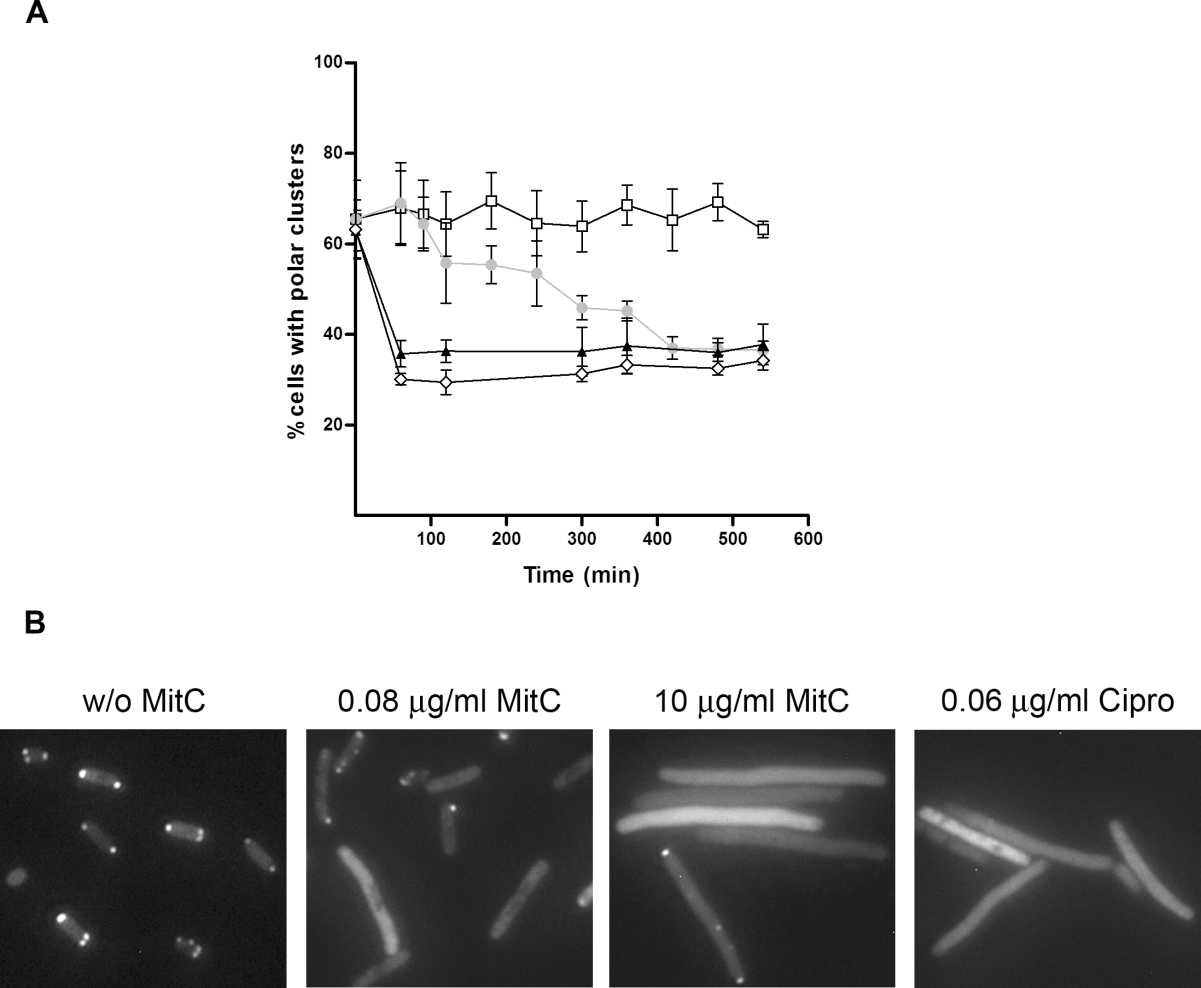
A) Evolution of the percentage of cells that developed polar chemoreceptor clusters during induction of the SOS system in a culture growing in liquid medium. The percentage of *S*. *enterica ΔcheR* cells harboring plasmid pUA1127, containing the inducible *eYFP*::*cheR* fusion, was quantified at several time points after the addition of either 0.08 or 10 μg mitomycin C/mL or 0.06 μg ciprofloxacin/mL concentration (● or ▲, respectively). Non-treated cells served as the control (□). The results are the mean of at least three independent imaging experiments. Error bars represent the standard deviation. B) Representative fluorescence microscopy images of cells treated for 300 min with either 0.08 or 10 μg mitomycin C/mL or 0.06 μg ciprofloxacin/mL. A control cell not treated with mitomycin C is shown as well.

**Fig 4:**
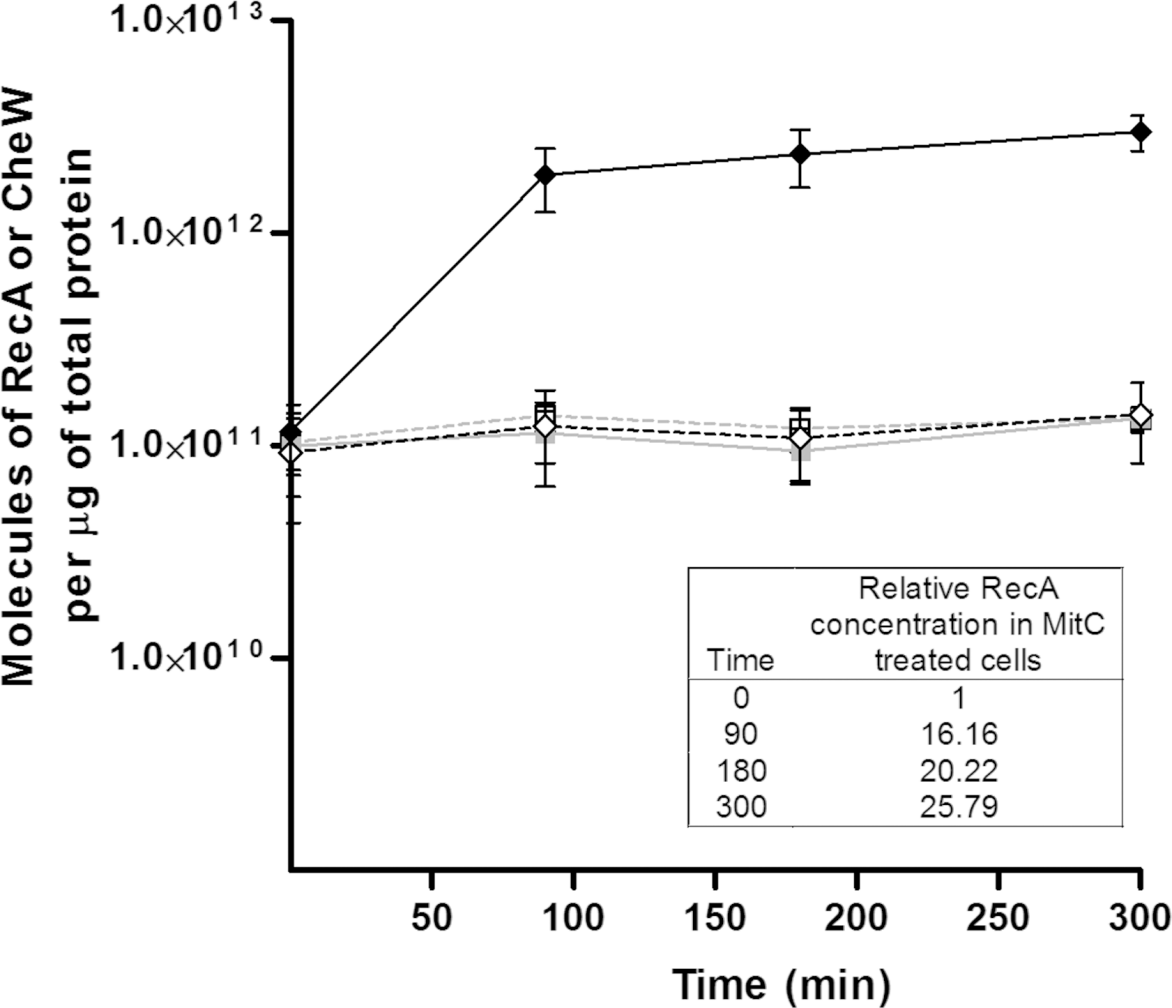
Concentration of RecA and CheW proteins in *S*. *enterica* mitomycin-C-treated cells growing in liquid medium. ELISA quantification of RecA (♦, continuous line) and CheW (■, continuous line) proteins of *S*. *enterica ΔcheR* cells harboring plasmid pUA1127 (eYFP::*cheR*) and treated with mitomycin C (0.08 μg/mL). The amounts of RecA (◇, discontinuous line) and CheW (□, discontinuous line) in a non-treated culture are also shown. The concentration is expressed as the number of RecA or CheW molecules per μg of total protein. The results are the mean of three independent experiments. Error bars represent the standard deviation. The relative RecA concentration (boxed) was calculated as the mean RecA concentration at each time point with respect to the mean initial RecA concentration [1.16 (±0.17) x 10^11^ molecules per μg of total protein].

Liquid cultures of *S*. *enterica ΔcheR cheW*::*FLAG/*pUA1127 were treated with two different mitomycin C concentrations. Polar cluster assembly and the concentrations of RecA and CheW during SOS response induction are shown in Fig 3 and Fig 4, respectively.

The kinetic assay of polar cluster assembly clearly indicated that the formation of chemoreceptor polar clusters was stable during bacterial population growth (Fig 3). Over time, in the absence of SOS system induction, about 65% of the cells contained polar clusters. This percentage is in concordance with data previously reported for *E*. *coli* cultures growing exponentially in liquid medium [53].

It should be noted that in *S*. *enterica* a higher percentage of cells with polar clusters are present when cells are growing on plates rather than those cultured in liquid medium (90% and 65%, respectively; Fig 2 and Fig 3). This may be due to the fact that, in plate cultures, the sampled cells were actively moving over the surface since they were those at the edge of the colony. In liquid cultures, the addition of mitomycin C caused a dose-dependent reduction in the number of cells containing chemoreceptor polar clusters (Fig 3). Specifically, after 300 min of treatment with 0.08 μg mitomycin C/mL, only about 45% of the cells contained polar clusters. This percentage continued to decrease for the next 2 h and then stabilized such that polar clusters were seen in only about 35% of the cells. In liquid cultures containing 10 μg mitomycin C/mL, the decrease in polar clusters occurred abruptly, with the minimum of 35% reached as early as after 60 min of incubation (Fig 3). Similar results were obtained when cells were treated with ciprofloxacin (Fig 3), another well-known SOS inducer [63], indicating that the polar clustering decrease is due to SOS activation and not specifically to mitomycin C treatment. Furthermore, all these results are also in agreement with the data obtained for cells swarming on plates (Fig 2). The similar decreases in polar clusters prompted by SOS response activation in both solid and liquid cultures suggests that mitomycin C reduces by half the number of polar-cluster-containing cells.

The absence of a mitomycin-C-induced variation in CheW over time was confirmed by ELISA (Fig 4). The amount of CheW in mitomycin-C-treated cells was similar to that in non-treated cells [mean = 1.17 (±0.17) × 10^11^ molecules CheW/μg total protein], which is in agreement with previous concentrations reported for *E*. *coli* [64]. Furthermore, the RecA concentration in non-SOS induced cells was nearly the same as that of CheW [mean = 1.16 (±0.21) × 10^11^ molecules RecA/μg total protein] and similar to that previously reported[43]. However, in the mitomycin-C-treated cultures, the RecA concentration rose quickly, increasing by about 25-fold after 300 min of treatment (Fig 2).

### Disconnection of the SOS response allows the recovery of cluster assembly

After determining that SOS response activation impairs polar cluster assembly, we analyzed the dynamics of clustering subsequent to the removal of mitomycin C, and therefore the cessation of DNA injury, from the cultures.

**Fig 5:**
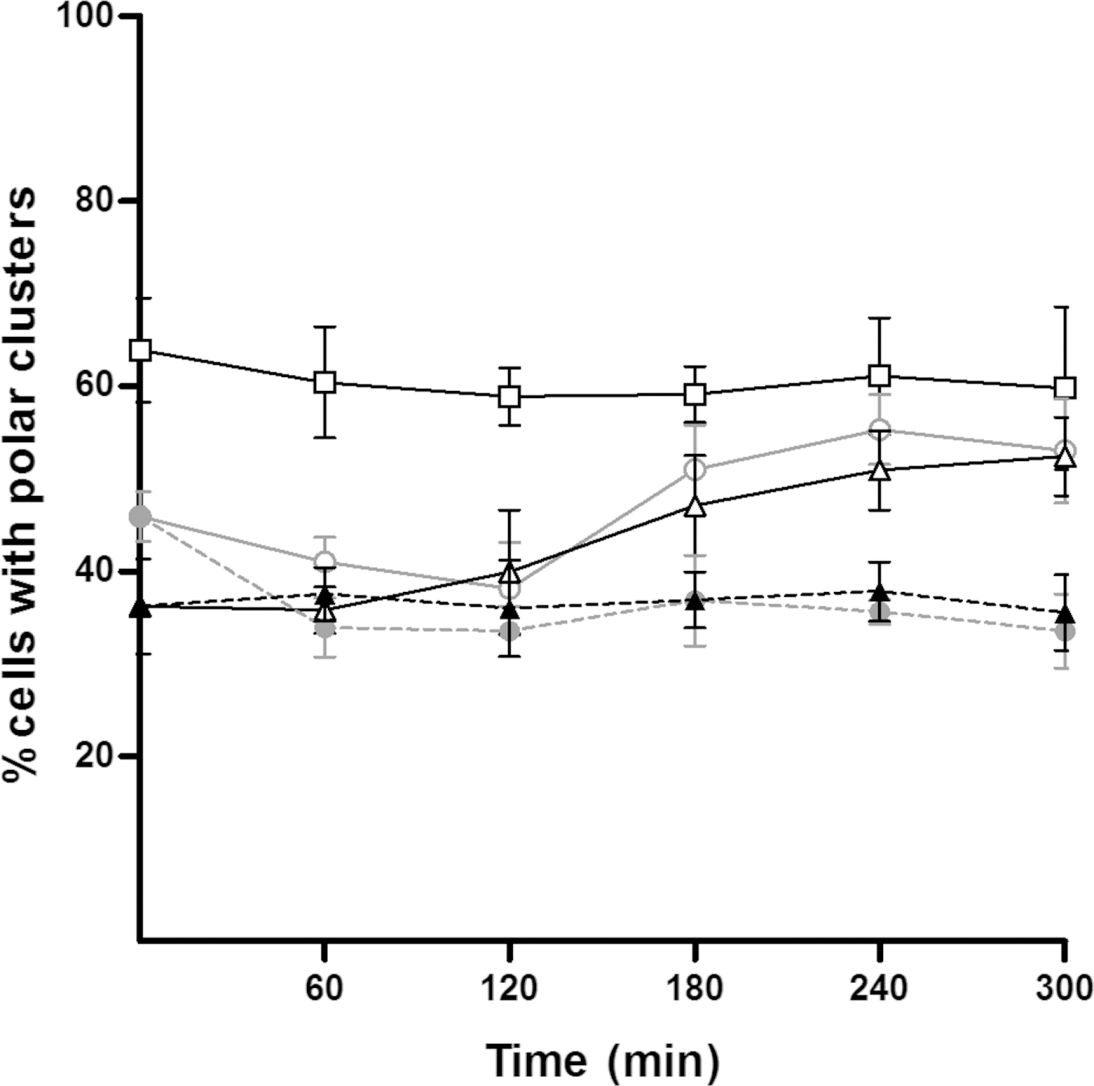
Evolution of the percentage of *S*. *enterica* cells that developed polar chemoreceptor clusters after cessation of SOS response induction in a culture growing in liquid medium. Cultures treated for 300 min with either 0.08 (○) or 10 (△) μg mitomycin C/mL were centrifuged to remove the inducer. Samples were periodically taken thereafter and the presence of polar clusters was determined. As controls, a non-treated culture (□) and two cultures treated again after centrifugation with either 0.08 (●, discontinuous line) or 10 (▲, discontinuous line) μg mitomycin C/mL are also shown. The results are the mean of three independent imaging experiments. Error bars represent the standard deviation.

**Fig 6:**
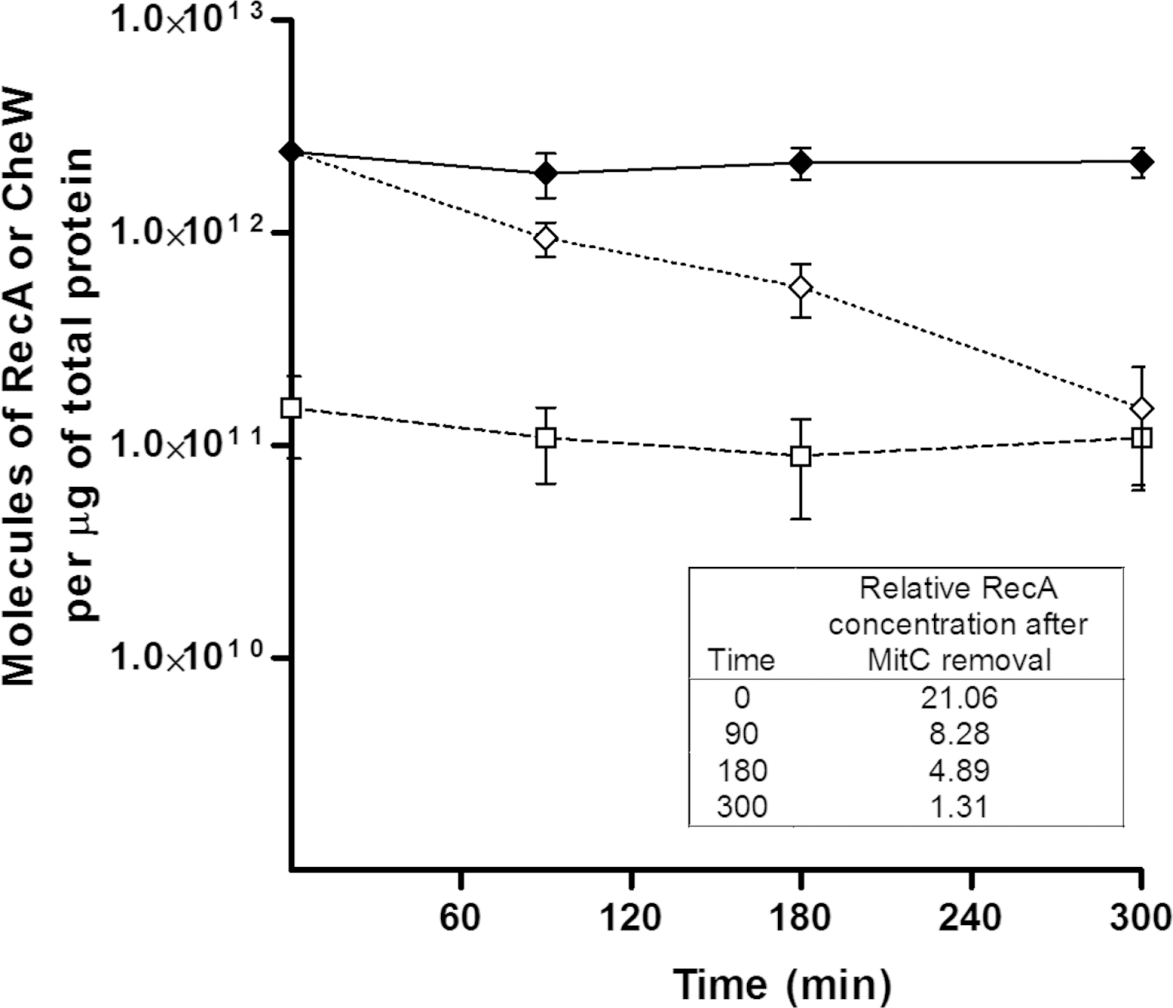
Concentration of RecA protein, as measured by ELISA, in *S*. *enterica ΔcheR* cells harboring plasmid pUA1127 (eYFP::*cheR*) and growing in liquid medium. Samples were taken periodically after the removal of mitomycin C from cultures pre-treated with 0.08 μg mitomycin C/mL for 300 min (◇, discontinuous dotted line). The amount of RecA of a non-treated culture (□, discontinuous line) and a continuously treated culture (◆, continuous line) served as controls. The RecA concentration is expressed as the number of RecA molecules per μg of total protein. The results are the mean of three independent experiments. Error bars represent the standard deviation. The relative RecA concentration after mitomycin C removal (boxed) was calculated as the mean RecA concentration at each time point with respect to the mean RecA concentration of the non-treated culture [1.13 (±0.25) x 10^11^ molecules per μg of total protein].

After treatment with either 0.08 or 10 μg mitomycin C/mL for 300 min, bacterial liquid cultures were centrifuged and resuspended in fresh medium no longer containing the SOS inducer. As shown in Fig 5, the percentage of cells with polar clusters progressively increased within 180 min after mitomycin C removal and after 240 min was close to the percentage in non-SOS-induced cultures regardless of the initial mitomycin C dose (Fig 5). Conversely, when mitomycin C treatment was maintained, the percentage of polar-cluster-containing cells remained at about 35% (Fig 5). At the same time, the concentration of RecA protein decreased and, like the percentage of cells containing polar clusters, gradually, returned to the basal level (Fig 5) by 300 min after mitomycin C removal (Fig 6). By contrast, mitomycin C removal had no effect on the CheW concentration, which remained the same as in non-treated cells (Fig 6). Thus, according to these observations, once SOS response activation ceases and basal RecA levels are reestablished, polar cluster assembly is restored, which implies that the SOS-mediated inhibitory effect is reversible.

### Impact of changes in the RecA/CheW balance

The above-reported results also implied a relationship between the intracellular RecA concentration and polar array assembly and, thereby, an effect on swarming motility. Accordingly, swarming of the *S*. *enterica lexA3*(Ind^−^) mutant was not affected by the presence of mitomycin C (Fig 1). Because an increase in CheW levels was previously shown to restore the swarming ability of a *recAo* strain [43], we analyzed the swarming behavior of *S*. *enterica* strains expressing different concentrations of either RecA or CheW proteins.

The *recA* gene was cloned into the pUA1108 overexpression vector, yielding the plasmid pUA1130, in which *recA* is under the control of an IPTG-inducible promoter (S1 Table). After pUA1130 was introduced into the *S*. *enterica cheW*::*FLAG* strain, both the swarming ability (expressed as the RSMI) [52] and the concentrations of RecA and CheW were determined in the presence and absence of IPTG. In these experiments, the samples were consistently taken from the edge of the plates (Fig A in S1 Fig, Table 1). As a control, the same strain but carrying the pUA1108 vector was studied under the same conditions (Fig A in S1 Fig, Table 1).

**Table 1:** Relationship between the RecA and CheW concentrations and the swarming ability of several *S*. *enterica* strains.

*S*. *enterica* strain	Overexpressed gene	IPTG (μM) treatment	RecA concentration (molecules/μg of total protein)^a^	CheW concentration (molecules/μg of total protein) ^a^	[RecA]/[CheW] ratio ^b^	Swarming phenotype^c^	RSMI^d^
		0					
		10					
*cheW*::*FLAG*/pUA1108^e^	none	20	2.03 (±0.60)x10^10^	2.15 (±0.21)x10^10^	0.94	++	1.00 (±0.08)
		30					
		40					
		50					
		0	9.86 (±0.26) x 10^10^		4.56	++	0.94 (±0.05)
*cheW*::*FLAG*/pUA1130 (P*tac*::*recA*)	*recA*	10	1.65 (±0.13) x 10^11^	2.16 (±0.41)x10^10^	7.64	+	0.44 (±0.08)
		20	3.39 (±0.34) x 10^11^		15.70	-	0.23 (±0.01)
		30	4.59 (±0.17) x 10^11^		21.25	-	0.16 (±0.03)
		0					
		10					
*recAo cheW*::*FLAG*/pUA1108^e^	*recA*	20	3.01 (±0.53)x10^12^	2.07 (±0.49)x10^10^	145.4	-	0.16 (±0.02)
		30					
		40					
		50					
		0		4.09 (±0.58) x 10^11^	7.80	-	0.14 (±0.01)
		10		1.73 (±0.20) x 10^12^	1.84	++	0.73 (±0.02)
*recAo cheW*::*FLAG/pUA113* (P*tac*::*cheW*)	*recA* and *cheW*	20	3.19 (±0.26)x10^12^	3.48 (±0.67) x 10^12^	0.91	++	1.07 (±0.01)
		30		6.01 (±0.85) x 10^12^	0.53	++	0.77 (±0.07)
		40		1.11 (±0.16) x 10^13^	0.28	-	0.31 (±0.14)
		50		1.21 (±0.11) x 10^13^	0.26	-	0.12 (±0.07)

^a^ The mean basal concentration of a given protein measured in at least three independent experiments is indicated in those cases in which its synthesis is not under IPTG control. The standard deviation is indicated in parentheses.

^b^The [RecA]/[CheW] ratio was calculated as the ratio of their respective concentrations at the indicated time point. When there was no difference in the protein concentration, the ratios were calculated using the mean values.

^c^(++) wild-type swarming ability. (+) reduced swarming ability, (-) no swarming ability.

^d^ The relative swarming colony motility index was calculated as the ratio between the colony diameter of the studied strain and that of the control strain under the same experimental conditions. The mean of at least three independent experiments is shown. The standard deviation is indicated in parentheses.

^e^ Expression vector that does not contain a gene fusion construct.

The presence of IPTG had no effect on the intracellular concentrations of RecA and CheW in *S*. *enterica* (pUA1108) cells (Table 1); rather, the concentrations [2.03 (±0.60) × 10^10^ and 2.15 (±0.21) × 10^10^ molecules of RecA and CheW/μg total protein, respectively] were proportional to those in non-treated cells grown in liquid culture (Fig 4). Nevertheless, the CheW and RecA concentrations were about 5-fold higher in cells growing in liquid cultures than on swarming plates. This is in agreement with previous reported data which described that the concentration of chemotaxis pathway proteins increases when cells are grown in nutrient-poor medium such as TB [65] herein used for visualizing chemoreceptor clusters [27]. Nevertheless this medium is not suitable for swarming assays that must be performed on LB-swarming plates [66].

The amount of RecA in cells carrying the *recA-*overexpressing plasmid (pUA1130) increased nearly 5-fold even in the absence of IPTG, as was expected because of the higher gene dosage. Nevertheless, this increase did not affect the swarming ability of these cells and the RSMI remained close to 1 (Table 1, S1 Fig). Only when *recA* expression was induced by IPTG, such that the RecA protein increased by about 8-fold with respect to the control strain, was swarming ability impaired. Moreover, swarming was totally abolished when the RecA concentration increased by >15-fold (Table 1, S1 Fig). These results are in complete agreement with those of the kinetic cluster assembly experiments (Fig 3 and Fig 4), in which a decrease in polar-cluster-containing cells was induced by increases in RecA concentrations up to 20-fold with respect to non-SOS-inducing conditions. They are also in concordance with the above-described results (Fig 5 and Fig 6) that once SOS response activation ceases, polar cluster assembly resumes when the RecA concentration is only about 5-fold higher than that of non-treated cells.

The data presented in Table 1 showed that the *recAo* strain carrying the *cheW* overexpression vector (pUA1131), in which RecA protein expression is 150-fold higher than that of the wild-type strain, is only able to swarm when the IPTG-mediated induction of CheW results in an increase of the protein to levels 80- to 280- fold with respect to wild type). The obtained results show that swarming ability is restored in the *recAo* strain when the RecA:CheW ratio is between 2 and 0.5; in all other cases, swarming is impaired (Table 1).

### Effect of a DNA-damaging compound gradient on swarming motility

During surface colonization driven by swarming motility, bacterial colonies may encounter DNA-damaging compounds, which would be present along a concentration gradient generated by their surface diffusion. To evaluate swarming behavior under these conditions, swarming assays were conducted in the presence of a mitomycin C gradient.

**Fig 7:**
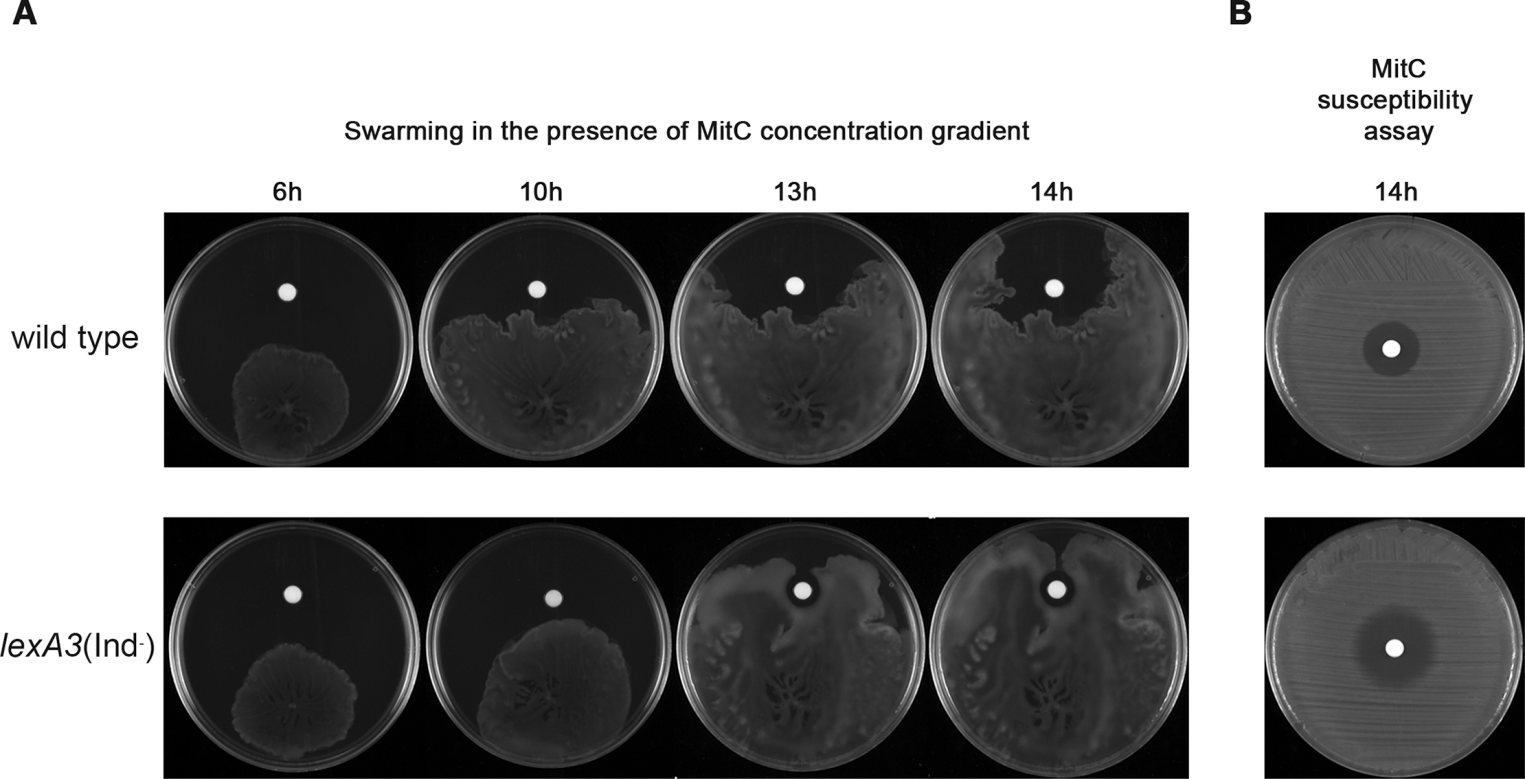
A) Swarming ability of either wild-type or *lexA3*(Ind^−^) strains in the presence of a mitomycin C concentration gradient. Swarming plates with a disk soaked with mitomycin C solution (2 mg/mL) were inoculated with the corresponding strain. Colony growth was followed by imaging the same representative swarming plate 6, 10, 13, and 14 h after plate inoculation. B) The susceptibility to mitomycin C of each strain was also evaluated. It must be noted that, as indicated in Material and Methods section, the swarming plates were point inoculated with the corresponding bacterial strain using a sterile toothpick while the susceptibility assays were carried out applying the bacterial inoculum using a sterile swab all over the plate surface.

Mitomycin C mediated-swarming inhibition was clearly observed by the wild type strain (Fig 7A). In fact, the swarming edge of wild type cells closest to the mitomycin-C-containing disk stopped but at other colony edges it proceeded, allowing colonization of the rest of the plate surface, where the mitomycin C concentration was low enough to be harmless. Nevertheless, no mitomycin C mediated-swarming inhibition was detected by the *lexA3*(Ind^−^) mutant (Fig 7A), which is unable to activate the SOS response. As expected, swarming was not affected when the disks were instead soaked in sterile water (data not shown). Since the sensitivity to mitomycin C (measured through determination of inhibitory growth halos) is higher in *lexA3*(Ind^−^) than in wild type cells (Fig 7B) the different behavior between these two strains in swarming plates (Fig 7A) must be attributed to the interference of the RecA protein increase upon this social motility during the SOS response induction. Further, the inhibition effect upon the *lexA3*(Ind^−^) strain growth around the mitomycin C disk while swarming (Fig 7A) was smaller than that generated for the same cells in the mitomycin C susceptibility assay (Fig 7B). This is in concordance with previous results in which high cell density and mobility diminishes the antibiotic effect against swarming bacteria [2]. All of these results indicate that the impairment of swarming by induction of the SOS system prevents the exposure of the cells to a lethal concentration of mitomycin C.

## Discussion

Activation of the SOS response in bacterial species such as *E*. *coli* and *S*. *enterica* prompts not only the error prone DNA repair pathway but also other cellular processes, such as the prevention of DNA degradation [67], the transitory inhibition of cell division [68] and respiration and sugar-related metabolic changes [69,70]. Some of these effects depend on the expression of specific chromosomal genes (e.g., the *sulA*-mediated inhibition of division [68]) whereas the basis of others is still unknown (cessation of cell respiration and the catabolism of sugars [69,70]). Other processes are the indirect consequence of SOS system induction. This is the case for the amplification of *recA* gene transcription, as the increased levels of RecA protein are able to bind to injured DNA blocking the access of DNAses such as RecBC [67]. This response together with the other functions of the SOS system contribute, both directly and indirectly, to ensuring the survival of bacteria populations in harmful environments.

The results reported herein show that the inhibition of bacterial colony motility over surfaces should be added to the pool of indirect phenomena associated with the SOS response. Our results demonstrate that, in *S*. *enterica*, SOS response activation impairs both chemoreceptor polar cluster assembly and consequently the swarming ability. These effects are due to the increase in the RecA concentration following SOS induction but not to other SOS-response-associated functions. The decrease of polar chemoreceptor clustering was observed in both the *recAo* and the *recAo lexA3*(Ind^−^) mutants whether in the absence or presence of SOS inducer (Fig 1 and Fig 2). The same results were obtained when RecA amplification was mediated by IPTG in a *S*. *enterica* strain carrying a P*tac recA* gene fusion (Table 1, S1 Fig). Swarming ability was totally abolished only when the RecA concentration increased, whether in response to DNA damage or IPTG addition indicating that no RecA activation is necessary for swarming inhibition (Fig 1 and Table 1).

Although CheW concentration does not change during SOS response induction (Figs 4 and 6), our data demonstrated a role for this protein in the RecA promoted modulation of swarming. The RecA and CheW proteins were present at similar concentrations in cells growing under non-SOS-inducing conditions. An increase in CheW levels restored the swarming ability of the *recAo* strain only if the levels of this protein were balanced with those of RecA (Table 1, S1 Fig), consistent with the interaction between RecA and CheW [32]. The RecA protein participates in multiple cellular functions, as DNA recombinase, SOS activation, and co-protease [36,71,72]. Thus, RecA interacts not only with other proteins but also with DNA. For this reason, ELISA results do not indicate the proportion of RecA that actually interacts with CheW *in vivo*. Studies on the specific stoichiometry of RecA and CheW would also shed light on the exact role of RecA protein in swarming control. For example, it could be that an increase in RecA prompts the titration of CheW, thus preventing chemoreceptor assembly and therefore also polar cluster array formation. However, this scenario is unlikely since *recA* defective mutants are also unable to swarm[32]. Another possibility is that RecA is a component of the chemoreceptor cluster such that, as described for CheW [27], high levels of the RecA protein interfere directly with chemoreceptor assembly. Further work is required to discern between these possibilities.

Another relevant aspect of the present work is the demonstration of the reversibility of the SOS response effect on polar cluster assembly (Fig 5). Specifically, under the conditions tested in this study, the normal percentage of polar-cluster-containing cells was reestablished 300 min after mitomycin C removal from the liquid cultures (Fig 5), as the amount of RecA decreased to its basal level because of the removal of this DNA damaging agent (Fig 6). Taking into account that the turnover time of RecA is about 15 min [73], the 300 min needed to reestablish the polar clusters and return both the SOS system and RecA to their basal levels probably reflected residual DNA damage, which continued to induce the SOS response until repair was completed (Fig 6).

**Fig 8:**
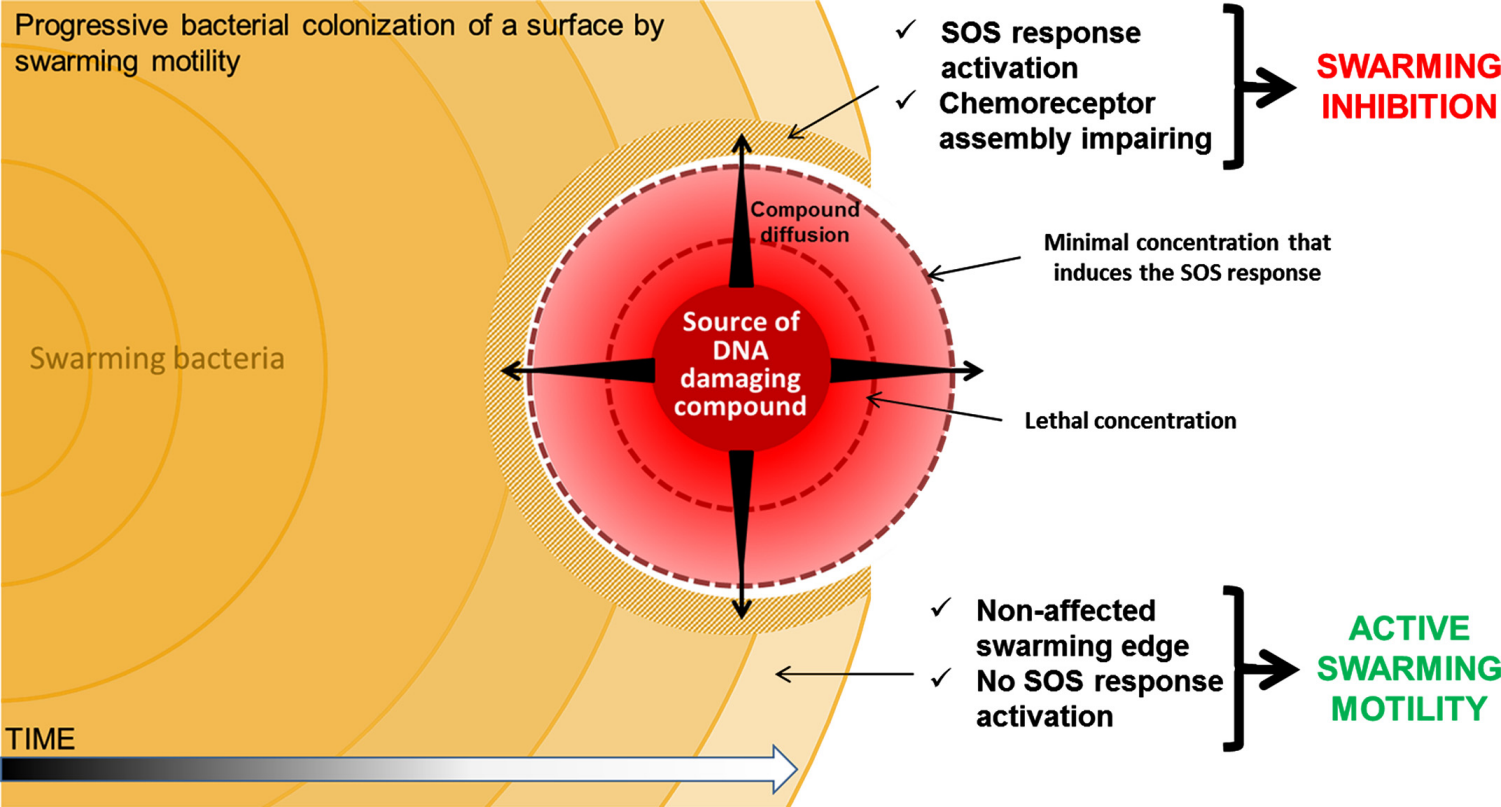
Proposed model for the control of swarming motility by the SOS response during bacterial surface colonization in the presence of a DNA damaging compound.

The reversibility of polar array assembly (Fig 5) and the swarming behavior in response to a concentration gradient of a SOS inducer (Fig 7A) are crucial aspects underlying the biological significance of the SOS response modulating swarming motility. Swarming control by the SOS response is summarized in Fig 8. The RecA protein, as the DNA-damage sensor, detects DNA injuries generated by the presence of SOS inducer compounds activating SOS response. Bacterial cells growing on surfaces will likely be exposed to a wide range of compounds, of either biological or chemical origin. By secreting toxic compounds such as antibiotics and bacteriocins, which diffuse though the growth surface, swarming bacterial colonies can impact neighboring cells of other species. However, once the SOS response is activated, the RecA concentration rises up quickly since *recA* is one of the first genes to be induced in the hierarchy of SOS activation [74–76]. This increase disturbs the equilibrium between this protein and CheW, which causes the cessation of swarming. In fact, when the bacterial colony edge is exposed to SOS-inducer, the swarming ability is impaired thus avoiding the exposure to higher concentrations of the injurious, and potentially lethal, compound. At the same time, the non-exposed edges of the colony continue to swarm and thus colonize those parts of the surface that are SOS inducer-free or contain a lower, non-injurious concentration of the DNA-damaging compound (Figs 7A and 8). In the case that the DNA-damaging source decrease or disappears, the repair of the DNA damage would restore polar cluster assembly and therefore also the colony swarming ability.

The chemotaxis pathway normally includes several receptors that detect either repellent or attractant compounds; for instance in *E*. *coli* at least five specific receptors have been described [77]. In contrast, the swarming response against DNA-damaging compounds reported herein is driven by a single signal (the SOS-response-mediated increase in RecA) that responds to a broad range of DNA-damaging agents. This general response mechanism is an advantage for bacterial cells, as it limits the number of genes required to one (*recA*). Taken together, these results further demonstrate the ability of bacterial cells to adapt their surface motility in response to the presence of direct or indirect DNA-damaging agents, by sensing these compounds via SOS system induction.

## Supporting Information

S1 Fig A) Swarming ability of the *S*. *enterica cheW*::FLAG strain harboring the *recA* expression vector (pUA1130) in the presence of increasing concentration of IPTG (0, 10, 20 and 30 μM). Representative images of swarming plates supplemented with the corresponding IPTG concentration are shown. As a control, colony swarming patterns of *S*. *enterica cheW*::FLAG carrying the expression vector (pUA1108) is shown. B) Representative plate images showing the recovery of swarming ability by the *S*. *enterica recAo cheW*::FLAG strain carrying the *cheW* expression vector (pUA1131) following the addition of IPTG. The swarming phenotype of this strain carrying the expression vector (pUA1108) is shown as a control.
(TIF)

S1 Table Bacterial strains and plasmids used in this work. (DOCX)
