# Supplementary figures and images for: SOS System Induction Inhibits the Assembly of Chemoreceptor Signaling Clusters in Salmonella enterica
Source: PLoS One. 2016 Jan 19;11(1):e0146685. doi: 10.1371/journal.pone.0146685 (PMC4718596; doi:10.1371/journal.pone.0146685)

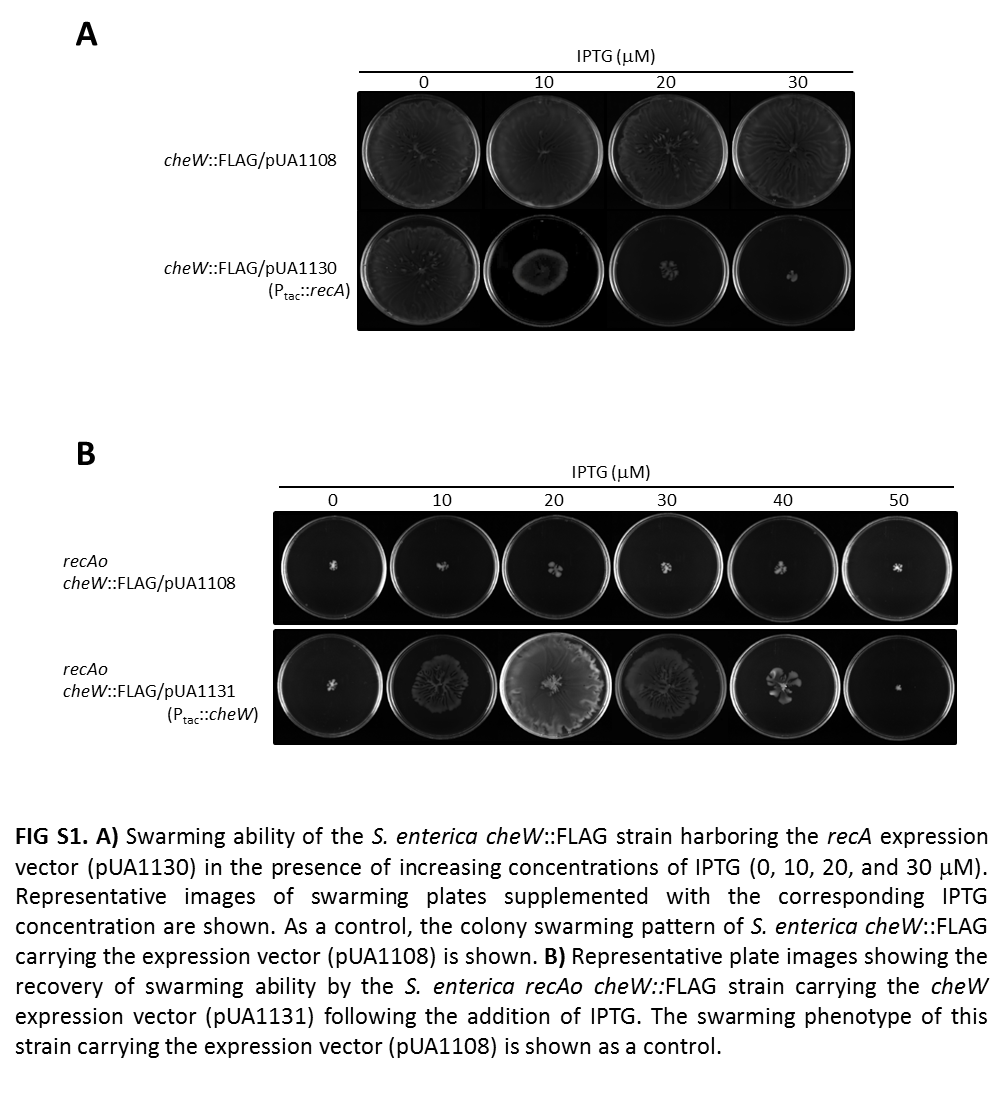

Supplement: S1 Fig — The swarming phenotype of this strain carrying the expression vector (pUA1108) is shown as a control. (TIF) [file pone.0146685.s001.tif]
