# Supplementary material for: SOS System Induction Inhibits the Assembly of Chemoreceptor Signaling Clusters in Salmonella enterica
Source: PLoS One. 2016 Jan 19;11(1):e0146685. doi: 10.1371/journal.pone.0146685 (PMC4718596; doi:10.1371/journal.pone.0146685)
Supplement: S1 Table — (DOCX) [file pone.0146685.s002.docx]

**S1Table.** Bacterial strains and plasmids used in this work

| **Strain or plasmid** | **Relevant characteristic(s)** | | **Source or reference** | |
| --- | --- | --- | --- | --- |
| **Strain** | |  | |  |
| DH5α | | *E. coli supE4 ΔlacU169 (φ80 ΔlacZ ΔM15) hsdR17, recA1, endA1, gyrA96, thi-1, relA1* | | Clontech |
| BL21 (DE3) pLysS | | *E. coli F^-^ dcm ompT lon hsdS(r_B_^-^m_B_^-^) galλ(DE3)* carrying pLysS plasmid, Cm^R^ | | Stratagene |
| ATCC14028 | | *S.* enterica Typhimurium wild type strain | | ATCC |
| UA1907 | | As ATCC14028 Ä*cheR*Ω*cat,* Cm^R^ | | [32] |
| UA1910 | | As ATCC14028 Ä*cheR* | | [32] |
| UA1876 | | As ATCC14028 but *recAo6869,* Km^R^ | | [63] |
| UA1822 | | As ATCC14028 but *lexA3*(Ind^-^), Cm^R^ | | [77] |
| UA1877 | | As UA1876 but *lexA3*(Ind^-^), Km^R^ Cm^R^ | | [63] |
| UA 1935 | | As ATCC14028 Ä*sulA* | | This work |
| UA1914 | | As UA1876 but Ä*cheR,* Cm^R^ | | This work |
| UA1932 | | As UA1822 but Ä*cheR,* Cm^R^ | | This work |
| UA1933 | | As UA1877 but Ä*cheR,* Km^R^ Cm^R^ | | This work |
| UA1936 | | As UA1935 but Ä*cheR,* Cm^R^ | | This work |
| UA1916 | | As ATCC14028 but *cheW::FLAG* | | This work |
| UA1917 | | As UA1876 *cheW::FLAG,* Cm^R^ | | This work |
| UA1934 | | As UA1910 but *cheW::FLAG,* Cm^R^ | | This work |
| **Plasmid** | |  | |  |
| pKOBEGA | | Vector containing the λ Red recombinase system,. Amp^r^, temperature sensitive | | [78] |
| pKD3 | | Vector carrying FRT-Cm construction, Amp^R^, Cm^R^ | | [48] |
| pCP20 | | Vector carrying FLP system, OriVts, Amp^R^ | | [48] |
| pGEX 4T-1 | | Expression vector carrying the P*tac* IPTG- inducible promoter and the *lacI^q^* gene; GST fusion tag, Amp^R^ | | Amersham Biosciences |
| pET15b | | Expression vector carrying the P*_T7_* IPTG- inducible promoter and the *lacI^q^* gene; 6X His fusion tag, Amp^R^ | | Novagene |
| pKO3 | | Vector for homologous recombination. OriVts, *sacB*, Cm^R^ | | [45] |
| pUA1108 | | pGEX 4T-1 derivative plasmid carrying only the P*tac* promoter and the *lacI^q^* gene; used as overexpression vector, Amp^R^ | | [32] |
| pUA1109 | | pUA1108 derivative containing the native *S.* Typhimurium *recA* gene under the control of the P*tac* promoter, Amp^R^. | | [32] |
| pUA1127 | | pUA1108 derivative vector carrying the *eYFP::cheR* fusion, Amp^R^ | | [32] |
| pUA1130 | | pUA1108 derivative containing the native *S.* Typhimurium *recA* gene under the control of the P*tac* promoter, Amp^R^. | | [32] |
| pUA1131 | | pUA1108 derivative overexpression vector carrying the *cheW*::FLAG gene | | [32] |
| pUA1121 | | pKO3 derivative carrying *cheW::FLAG* fusion, Cm^R^ | | This work |
| pUA1125 | | pGEX4T1-1 carrying the *recA* gene. Overexpression plasmid for RecA purification, Amp^R^ | | This work |
| pUA1132 | | pET15b carrying the *cheW::FLAG* fusion. Overexpression plasmid for CheW::FLAG purification, Amp^R^ | | This work |
